# Severe Early-Onset Obesity Due to Bioinactive Leptin Caused by a p.N103K Mutation in the Leptin Gene

**DOI:** 10.1210/jc.2015-2263

**Published:** 2015-07-17

**Authors:** Martin Wabitsch, Jan-Bernd Funcke, Julia von Schnurbein, Friederike Denzer, Georgia Lahr, Inas Mazen, Mona El-Gammal, Christian Denzer, Anja Moss, Klaus-Michael Debatin, Peter Gierschik, Vanisha Mistry, Julia M. Keogh, I. Sadaf Farooqi, Barbara Moepps, Pamela Fischer-Posovszky

**Affiliations:** Division of Pediatric Endocrinology and Diabetes (M.W., J.-B.F., J.v.S., F.D., C.D., A.M., P.F.-P.), and Department of Pediatrics and Adolescent Medicine (G.L., K.-M.D.), University Medical Center Ulm, 89075 Ulm, Germany; Clinical Genetics Department (I.M., M.E.), National Research Center, Cairo 12311, Egypt; Institute of Pharmacology and Toxicology (P.G., B.M.), University Medical Center Ulm, 89081 Ulm, Germany; and University of Cambridge Metabolic Research Laboratories (V.M., J.M.K., I.S.F.), Wellcome Trust-MRC Institute of Metabolic Science, Addenbrooke's Hospital, Cambridge CB2 0QQ, United Kingdom

## Abstract

**Context:**

Congenital leptin deficiency is a very rare cause of severe early-onset obesity. We recently characterized a mutation in the leptin gene (p.D100Y), which was associated with detectable leptin levels and bioinactivity of the hormone.

**Case Description:**

We now describe two siblings, a 9-year-old girl and a 6-year-old boy with severe early-onset obesity and hyperphagia, both homozygous for a c.309C>A substitution in the leptin gene leading to a p.N103K amino acid exchange in the protein and detectable circulating levels of leptin. In vitro experiments in a heterologous cell system demonstrated that the mutated protein was biologically inactive. Treatment with sc recombinant human leptin led to rapid improvement of eating behavior and weight loss.

**Conclusions:**

Sequencing of the leptin gene may need to be considered in hyperphagic, severely obese children with detectable levels of circulating leptin.

Congenital leptin deficiency is characterized by hyperphagia and severe early-onset obesity, along with metabolic and endocrine derangements and in some cases also immunological alterations (1–3). The disease is caused by mutations in the *LEP* gene typically leading to defects in protein synthesis or secretion, and therefore to the absence or very low levels of the hormone in the circulation (1, 2). Recently, we described the first case of functional leptin deficiency (4). This entity is characterized by detectable immunoreactive levels of circulating leptin, but bioinactivity of the hormone due to defective receptor binding (4). We now describe two additional cases of functional leptin deficiency due to a c.309C>A substitution in the *LEP* gene resulting in an asparagine to lysine amino acid exchange at position 103 of the protein (p.N103K).

## Subjects and Methods

### Leptin sequencing and ELISA

All human studies were conducted according to the principles outlined in the Declaration of Helsinki and after approval by local ethical committees. All individuals or their parents (for children) gave written informed consent. Severely obese patients from the Genetics of Obesity Study (GOOS) cohort (5) were sequenced for mutations in leptin gene (*LEP*) as reported previously (3).

Leptin concentrations were measured by ELISA using kits from IBL, with a detection limit of 1 ng/mL, interassay coefficient of variation (CV) of 8.7–11.6%, and intra-assay CV of 6.0–6.9%; and from Assaypro, with a detection limit of approximately 0.12 ng/mL, interassay CV of 7.0%, and intra-assay CV of 4.7%. Leptin concentrations in cell culture media were measured using a kit from BioVendor with a detection limit of 0.17 ng/mL, interassay CV of 7.5%, and intra-assay CV of 9.2%, respectively.

### Transfection and functional studies

Plasmid vectors encoding wild-type and p.N103K leptin and corresponding fluorescent fusion proteins were generated as described earlier (4). Leptin-containing cell culture supernatants were prepared by transfection of HEK293 cells as described (4, 6). For Western blot analysis of leptin production and secretion, a rabbit polyclonal antileptin antibody (BioVendor) was used.

For signaling studies, HEK293-hLR-FLAG cells transiently overexpressing the human leptin receptor (hLR-FLAG, kindly provided by Jan Tavernier, University of Ghent, Belgium) were treated with leptin-containing supernatants (adjusted to 30 ng/mL leptin). Cell lysates were subjected to Western blot analysis as described (4).

For internalization studies, HEK293-hLR-FLAG cells were treated with leptin-mCherry-containing supernatants (adjusted to 30 ng/mL leptin). The cells were fixed with paraformaldehyde, counterstained with Hoechst 33342 (trihydrochloride-trihydrate; Life Technologies), and subjected to fluorescence analysis on an Olympus IX-70 microscope (Olympus).

## Results

### Case history

**Figure 1.**
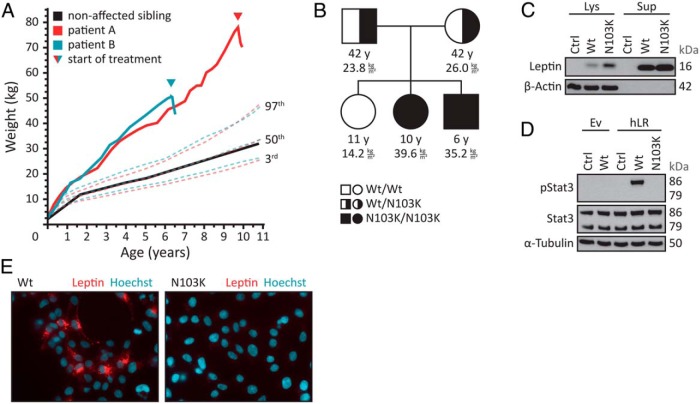
Bioinactivity due to a p.N103K mutation in leptin. A, Body weight curves of the patients (boy, blue line; girl, red line) and the healthy, normal-weight sister (black line) compared to body weight percentiles for boys (blue dotted lines) and girls (red dotted lines). The triangle indicates the start of treatment. B, Pedigree indicating the mutation status of the parents and their three children. The BMI is given in kilograms per square meter, along with the corresponding age in years. C, HEK293 cells were transfected with empty vector (Ctrl) or vector encoding wild-type (Wt) or p.N103K (N103K) leptin. After 48 hours, media supernatants (Sup) were collected, cell lysates (Lys) were prepared, and leptin immunoreactivity was examined by Western blot analysis. β-Actin served as a loading control. One representative experiment out of three performed is shown. D, HEK293 cells were transfected with either empty vector (Ev) or vector encoding the human leptin receptor (hLR). Cells were treated with media supernatants from HEK293 cells transfected with empty vector (Ctrl) or vector encoding wild-type (Wt) or p.N103K (N103K) leptin for 15 minutes. Concentrations of wild-type and p.N103K leptin were adjusted to 30 ng/mL. Cell lysates were prepared and subjected to Western blot analysis using pStat3 and Stat3 antibodies. α-Tubulin served as a loading control. One representative experiment out of three performed is shown. E, HEK293 cells were transfected with vector encoding the human leptin receptor. Cells were treated with media supernatants from HEK293 cells transfected with vector encoding mCherry-labeled wild-type (Wt) or p.N103K (N103K) leptin for 45 minutes. Concentrations of wild-type and p.N103K leptin were adjusted to 30 ng/mL. The cells were fixed, the nuclei were stained with Hoechst 33342 (Hoechst), and the cells were analyzed by fluorescence microscopy. Blue, nuclei; red, mCherry-labeled leptin. One representative experiment out of three performed is shown.

The patients are siblings, the second and third children of two healthy, nonobese Caucasian (German) parents without known consanguinity. Patient A, the second-born girl, was delivered by cesarean section. Birth weight and height were normal. After 4 weeks of full breastfeeding, she became clinically conspicuous by insatiable appetite with the introduction of bottle-feeding at the age of 5 weeks. She rapidly gained weight and developed severe obesity (Figure 1A). Circulating levels of leptin were high (>50 ng/mL). Intensive outpatient and inpatient measures aiming at influencing eating behavior as well as the mother-child interaction were made, but with marginal success. An experimental treatment trial with methylphenidate for 13 months, with a maximal dose of 60 mg/d starting at the age of 7 years, resulted in a reduction of the body mass index (BMI) from 35.6 to 34.8 kg/m^2^. Ultimately, however, none of the interventions could influence the dramatic weight development. Information on anthropometric, endocrine, and metabolic parameters is summarized in Table 1.

**Table 1.** Anthropometric, Endocrine, and Metabolic Characteristics of Patients Before and After the Start of Treatment With Metreleptin

Parameter/Time of Treatment	Patient A				Patient B			
	Day 0	Day 4	Day 9	Week 8	Day 0	Day 4	Day 9	Week 8
Age, mo	117	117	117	119	76	76	76	78
Weight, kg	77.9	78.1	76.7	71.7	50.4	51.3	50.6	46.6
BMI, kg/m^2^	39.6	39.7	39.0	35.8	35.2	35.8	35.3	31.7
BMI z-score	3.5	3.5	3.5	3.3	4.1	4.1	4.1	3.8
Leptin, ng/mL	59.7	56.2	46.9	64	74.6	nd	56.8	20.4
Insulin, mU/L	32.5	30.6	51.1	22.7	19.8	13.8	8.8	10.8
C-peptide, ng/mL	3.8	3.6	4.6	3	3	2.8	1.8	2.2
Triglycerides, mmol/L	1.4	1.5	1.6	1.5	0.8	1.2	0.4	0.9
ALT, U/L	20	18	21	35	16	32	41	37
AST, U/L	22	25	27	35	25	36	53	43
GGT, U/L	15	14	nd	11	17	19	nd	30
Cortisol, μg/dL	4.8	5.8	5.8	16.3	8.8	8.5	9.5	5.1
IGF-1, ng/mL	134	129	133	153	41	54	54	70
IGFBP-3, ng/mL	3360	3470	3950	3590	1830	2450	2400	2560
LH, U/L	<0.1	<0.1	nd	<0.1	<0.1	<0.1	nd	<0.1
FSH, U/L	0.3	0.3	nd	1.05	0.6	nd	nd	1.8

Abbreviations: ALT, alanine transaminase; AST, aspartate transaminase; GGT, γ-glutamyl transpeptidase; IGFBP-3, IGF binding protein 3; nd, not determined.

The third-born boy displayed the same symptoms as the second-born child (Table 1). Born with normal weight, he rapidly showed marked hyperphagia and pronounced weight gain (Figure 1A) with high levels of serum leptin (>70 ng/mL). The first-born girl showed normal weight development (Figure 1, A and B).

On the basis of our recent description of the first case of congenital functional leptin deficiency (4), we had the suspicion of biological inactive leptin also in these two siblings. Analysis of the *LEP* gene sequence showed a homozygous cytosine to adenine base substitution (transversion) in exon 3 (c.309C>A), which resulted in an asparagine to lysine amino acid exchange in the protein (p.N103K). Both parents were heterozygous carriers of the mutation, whereas the lean sibling was homozygous for the wild-type allele (Figure 1B). Sequencing of an additional 290 children with severe early-onset obesity with detectable leptin levels from the Genetics of Obesity Study (GOOS) did not reveal additional mutations in *LEP*. Because this cohort included patients from consanguineous families and with clinical features strongly suggestive of the disorder, these findings suggest that mutations resulting in a bioinactive form of leptin are a very rare cause of severe, early-onset obesity.

Secretion studies in HEK293 cells overexpressing either the wild type or the mutant leptin demonstrated that the p.N103K variant is indeed released into the cell culture medium (Figure 1C). However, whereas the wild-type leptin was able to induce the phosphorylation of Stat3 in HEK293 cells overexpressing the human leptin receptor, the p.N103K mutant was unable to do so (Figure 1D). Likewise, the mutant mCherry-tagged leptin did not bind to or cause internalization of the leptin receptor, whereas the wild-type hormone exerted these functions (Figure 1E). This set of experiments clearly demonstrates that p.N103K leptin mutant is secreted, but is not functional. As expected, treatment of both children with metreleptin injection of 0.03 mg per kilogram of lean body weight per day led to an improvement of hyperphagia, satiety after consumption of normal amounts of food, and subsequent weight loss (Figure 1A).

## Discussion

This case report demonstrates that functional leptin deficiency should be considered in children with uncontrollable hyperphagia and rapid weight gain in early childhood. Although measuring serum levels of leptin is appropriate to diagnose classical congenital leptin deficiency, which is characterized by the absence or very low levels of the hormone in the circulation, sequence analysis of the *LEP* gene revealed a small subset of patients with a bioinactive form of leptin, as described earlier for the p.D100Y (4) and herein for the p.N103K mutant.

From a structural point of view, the p.N103K substitution does not appear to alter the overall structure of helix C of leptin. Similar to the side chains of p.D100 and p.Y100, the side chains of p.N103 and p.K103 protrude from helix C of leptin to the surface of the leptin binding domain (LBD) of the human leptin receptor. At the point of contact to leptin p.N103, the binding site on LBD is made up of a hydrophobic IFLL cluster (amino acids 503 to 506 of the human leptin receptor, Uniprot ID: P48357–1). The binding site on LBD can accommodate the neutral carboxamide side chain of p.N103. The side chain of p.K103, however, is both longer by about 2.6 Å (7) and positively charged at physiological pH. Thus, it is likely that the hydrophobic IFLL pocket on LBD does not accommodate p.K103 as a substitute of p.N103. The p.N103K mutation described here has already been reported in two obese patients from Egypt, but it was described to be associated with low levels of circulating leptin (8). We first suspected that the ELISA kit used in that study might have been unable to recognize the mutant protein. This was, however, excluded by measuring patient serum and media supernatants from HEK293 cells overexpressing the p.N103K leptin with the ELISA kit used in the original study, which yielded concentrations comparable to those measured with other kits (>50 ng/mL leptin for both patients). Interestingly, the biological activity of p.N103K leptin has been addressed earlier (9). In line with our findings, Niv-Spector et al (9) reported a reduced binding affinity of the mutant protein to the LBD of the human leptin receptor. Our data provide in vivo proof that the p.N103K mutation causes obesity due to biological inactivity, but in the presence of high circulating levels of the mutant leptin hormone. Taken together, our study once again illustrates bioinactivity of leptin as a cause of pharmacologically treatable, early-onset, severe obesity.
